# Flexible All-organic, All-solution Processed Thin Film Transistor Array with Ultrashort Channel

**DOI:** 10.1038/srep29055

**Published:** 2016-07-05

**Authors:** Wei Xu, Zhanhao Hu, Huimin Liu, Linfeng Lan, Junbiao Peng, Jian Wang, Yong Cao

**Affiliations:** 1 Institute of Polymer Optoelectronic Materials and Devices, State Key Laboratory of Luminescent Materials and Devices, South China University of Technology, Guangzhou 510640, P. R. China

## Abstract

Shrinking the device dimension has long been the pursuit of the semiconductor industry to increase the device density and operation speed. In the application of thin film transistors (TFTs), all-organic TFT arrays made by all-solution process are desired for low cost and flexible electronics. One of the greatest challenges is how to achieve ultrashort channel through a cost-effective method. In our study, ultrashort-channel devices are demonstrated by direct inkjet printing conducting polymer as source/drain and gate electrodes without any complicated substrate’s pre-patterning process. By modifying the substrate’s wettability, the conducting polymer’s contact line is pinned during drying process which makes the channel length well-controlled. An organic TFT array of 200 devices with 2 *μ*m channel length is fabricated on flexible substrate through all-solution process. The simple and scalable process to fabricate high resolution organic transistor array offers a low cost approach in the development of flexible and wearable electronics.

Organic thin-film transistors (OTFTs) have attracted great attention for a wide range of applications, such as flexible displays^1^,^2^, sensors^3^, radio frequency identification tags^4^, low-cost memories^5^, and microprocessors^6^, due to their unique properties of excellent mechanical flexibility and process advantages of being compatible with high throughput and low-budget printing processes^7^. Over the years, significant efforts have been made to develop high carrier mobility, chemically and physically stable organic semiconductor materials with the reported device performance of OTFTs already better than that of amorphous silicon devices^8^,^9^,^10^,^11^. However, the fabrication of OTFT devices generally requires thermal evaporation through masks to deposit metal electrodes and the semiconducting layer in vacuum^1^,^2^,^3^,^4^,^5^,^6^,^7^,^8^,^9^,^10^,^11^,^12^,^13^,^14^. The process is known to be costly and difficult to scale up. To fully exploit the potentials of organic materials in the applications of low cost and flexible electronics, all-organic TFTs made by all-solution process are desired^15^.

Various techniques have been developed to fabricate all-organic TFTs. Conventional photolithography, which is the standard technique in inorganic semiconductor industry, has been explored for patterning polymer electrodes in bottom contact OTFTs. For example, Halik *et al*. fabricated all-organic pentacene TFTs on flexible polymeric substrates with photolithography-patterned PEDOT:PSS contacts^16^. A similar photo-induced patterning approach, named photochemical patterning, was developed by Gelinck *et al*. They demonstrated fully-solution-processed and all-polymer integrated circuits with conductive polyaniline electrodes defined by photochemical conversion through a shadow mask^17^. Although photolithography is a mature process and capable of high resolution patterning, its application in patterning OTFTs is rather limited due to degradation of organic semiconductors upon exposure of the etching chemicals.

Screen printing, which is suitable for large scale production and much cheaper than photolithography, has been exploited in OTFTs much earlier than photolithography. Garnier *et al*. demonstrated the first all-organic TFTs back in 1994 of which the source, drain and gate electrodes were all screen-printed from a graphite based ink, while the small molecular semiconductor was thermally evaporated^18^. Most recently, Hyun *et al*. adopted a photolithography-patterned silicon sheet as the stencil to improve the resolution of screen printing^19^. All-solution processed, electrolyte gated transistors were fabricated with screen-printed graphene source and drain (S/D), and an aerosol-jet printed poly(3,4-ethylenedioxythiophene) polystyrene sulfonate (PEDOT:PSS) gate. However, the obtained channel length of 90 *μ*m is not narrow enough to meet the demand for large drive current and high switching speed.

Microcontact printing, which transfers patterns from stamps, can achieve a resolution as high as that of photolithography while retaining low cost. Parashkov *et al*. employed the process to pattern an electron-transfer blocking layer on a gold plate to realize selective electro-polymerization of PEDOT:PSS S/D^20^. The electrodes were then transferred onto a polyimide substrate where pentacene and polyvinyl alcohol (PVA) were deposited as the semiconductor and dielectric layer. The PEDOT:PSS gate was screen printed. The final channel length is as small as 10 *μ*m. However, the costly metal deposition in vacuum for preparing the electro-polymerization template was required to deposit the electrodes.

Another simple and cost-effective patterning technique is inkjet printing. The advantages of its fully additive feature, flexibility in pattern design, and low material consumption, make inkjet printing an attractive deposition technique in OTFT fabrication. The patterning resolution of inkjet printing, however, is generally tens of micrometers owing to the difficulty in controlling the droplet size and ink-spreading on substrates. To overcome the resolution limitation, Sirringhaus *et al*. demonstrated an approach by depositing the functional ink onto a substrate containing a predefined surface-energy pattern that was able to split the deposited ink droplets to form a narrow gap^15^,^21^. Sub-micrometer channel length was achieved by inkjet printing PEDOT:PSS ink. The process requires photolithography or electron-beam lithography to prepare the hydrophobic strips. To further exploit the direct-writing capability by inkjet printing, the authors developed a lithography-free nanopatterning technique in which a self-assembled surface layer induced the dewetting of PEDOT:PSS resulting in sub-micrometer size channels^22^. However, the additional surface layer sometimes causes defects at electrode contacts, and the dewetting process is time consuming.

All aforementioned techniques require multi-step procedures to pattern organic electrodes in order to achieve the narrow channel length. In our contribution, we develop a direct-writing technique to pattern organic electrodes with high resolution by inkjet printing. All-organic and all-solution processed TFT arrays were fabricated on a flexible substrate. By modifying the surface wettability of the polyethylene terephthalate (PET) surface with PVA, a channel length around 2 *μ*m defined by PEDOT:PSS electrodes was achieved. To the best of our knowledge, this is the highest resolution ever reported for directly inkjet-printed electrodes without any complicated substrate pre-patterning process. A 10 by 20 OTFT array were fabricated across a 3 cm × 3 cm flexible substrate showing good uniformity and high yield. Our study demonstrates that by carefully controlling ink spreading on substrates, high resolution OTFTs are achievable by direct inkjet printing.

## Results

**Figure 1:**
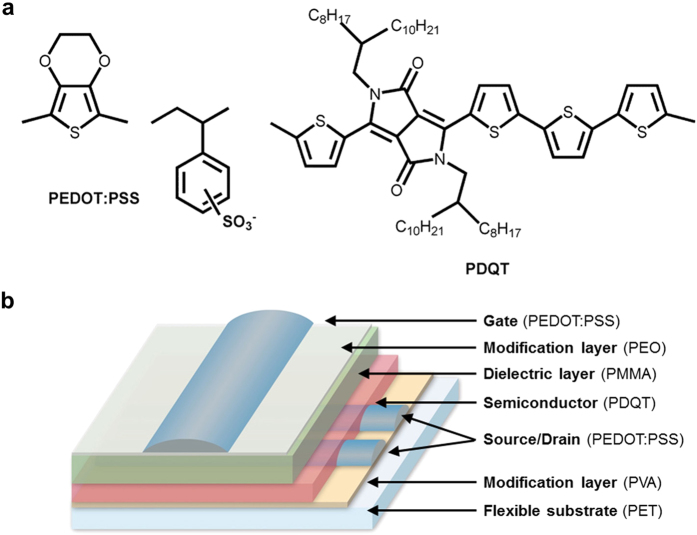
OTFT structure. (**a**) Molecular structures of PEDOT:PSS and PDQT. (**b**) Device structure of all-organic, all-solution processed OTFTs with inkjet-printed PEDOT:PSS electrodes on flexible substrate.

To fabricate OTFTs on a flexible substrate, a 200 *μ*m thick PET foil first went through 20 mins oxygen-plasma treatment to increase the surface energy. PVA in deionized water (20 mg/mL) was spin-coated onto PET film to form a 50 nm buffer layer to modify the PET surface. On top of the PVA surface modification layer, S/D electrodes were fabricated by line printing conducting polymer PEDOT:PSS^23^. The organic semiconductor layer was deposited by spin-coating poly [3,6-bis(40-dodecyl[2,20]bithiophenyl-5-yl)-2,5-bis(2-hexyldecyl)-2,5-dihydropyrrolo[3,4-c]pyrrole-1,4-dione] (PDQT) in chloroform (6 mg/mL). The thickness of the active layer is about 60 nm. On top of the active layer, a 500 nm film of polymethylmethacrylate (PMMA) was spin-coated from its n-butyl acetate solution (50 mg/mL) as the gate dielectric layer. An additional ultrathin polyethylene glycol (PEO) layer was spin-coated from a 5 mg/mL solution in methanol to modify the PMMA surface to enhance its wettability for the subsequent PEDOT:PSS gate printing, which was laid perpendicular to the S/D lines. The top-gate OTFT structure is schematically illustrated in Fig. 1. All the processes are solution-based, and all the processes are carried out in air at room temperature.

Reducing the channel length to achieve high device integration density, high operation speed and low power consumption is critical in OTFT development. Grau *et al*. reported gravure-printed OTFTs with 5 *μ*m channel length^24^. Yoshimura *et al*. achieved 4 *μ*m channel length by inkjet printing silver inks^25^. Fukuda *et al*., taking advantage of the high-resolution off-set printing and the their customized silver ink, reached a submicrometer channel length of 0.6 *μ*m^26^. However, the studies to achieve narrow channel length all used metal inks. Though direct inkjet-printing conducting polymers (mainly PEDOT:PSS) as S/D electrodes in OTFT devices without pre-patterning substrate has been demonstrated^27^, the typical channel length is between 20 to 50 *μ*m owing to the ink spreading on substrates and droplet positioning error^15^. To overcome the problem, we propose here that by modifying the surface energy of the substrate with a hydrophilic and dissolvable layer, namely, the PVA buffer layer, accurate PEDOT:PSS patterns of short channels becomes possible due to the contact line pinning effect and excellent film morphology.

**Figure 2:**
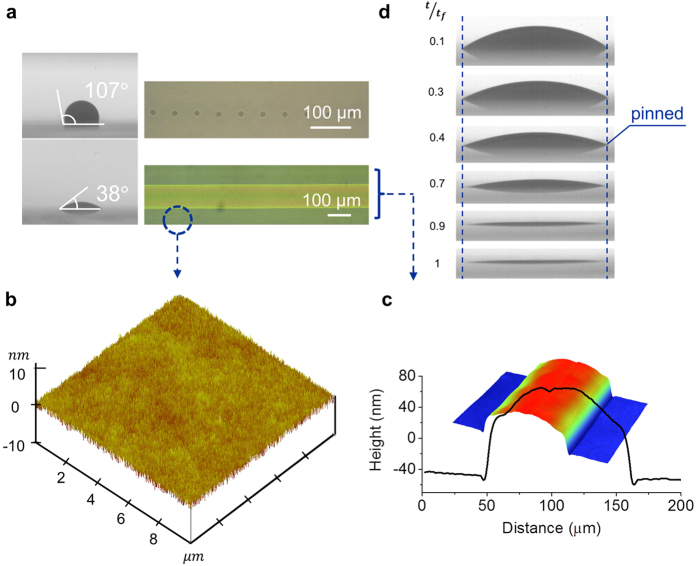
Printed PEDOT:PSS lines on the PVA modified substrate. (**a**) The contact angle of PEDOT:PSS on PET surface is 107°. Line printing on the substrate results in isolated islands due to the small solid surface energy. After PVA modification, PEDOT:PSS contact angle becomes 38°. A continuous line is formed by line printing PEDOT:PSS. (**b**) The AFM picture shows the smooth PVA-modified PET surface. (**c**) The three-dimensional white light interference microscopic picture and the cross-sectional profile of the printed PEODT:PSS line on PVA-modified PET. (**d**) Sequential picture of PEDOT:PSS drying process on PVA-modified PET. *t*_*f*_ is the total drying time.

To obtain ultrashort channel, the S/D electrodes’ patterning process is crucial. During the inkjet printing process, upon striking the substrate, the PEDOT:PSS droplet spreads first as driven by the kinetic energy. Afterwards, it shrinks because of the surface tension, and finally dries to form a stable shape^28^. After inkjet printing PEDOT:PSS in the polar solvent (water) on a non-polar PET surface, due to the high contact angle of 107°, the consecutively printed PEDOT:PSS droplets can’t form a continuous line. Instead, isolated islands were formed, as demonstrated in Fig. 2a. To increase the hydrophilicity of the substrate, PVA was spin-coated to modify the PET surface. The atomic force microscopy (AFM) image in Fig. 2b shows the PVA has a very smooth surface with a root mean square (RMS) roughness of about 1.2 nm. On PVA, the contact angle of PEDOT:PSS was reduced to 38°. As the result, a uniform electrode line was formed by the consecutively printed PEDOT:PSS droplets as shown in Fig. 2a. The three-dimensional picture from the white light interference microscope shows a relative smooth surface of the PEDOT:PSS line free of the coffee ring (Fig. 2c). It is determined by the cross-section profile that the line width is about 106 *μ*m, and the line thickness is about 100 nm.

A common practice to increase the substrate surface energy is UV/ozone or oxygen plasma treatment^29^,^30^. However, the wettability provided by the treatments is not stable in air^31^,^32^. With PVA film, the hydrophilic layer guarantees the uniform coating of the PEDOT:PSS electrode array across a large area during the printing process without any wettability deterioration.

Moreover, the PVA layer can pin the contact line during PEDOT:PSS film formation. Figure 2d shows the drying process of the PEDOT:PSS droplets on PVA/PET substrate. The total drying time *t*_*f*_ was 600 s. The contact line (as denoted by the dash lines) was strongly pinned due to the dissolution of PVA underneath the PEDOT:PSS droplet, and the contact line did not withdraw during the whole drying process as shown at *t* = 60 s, 180 s, 240 s, 420 s , 540 s, and 600 s, respectively. In our early work, PEDOT:PSS was inkjet printed on an oxygen-plasma treated PMMA surface^33^. The contact line withdrew during PEDOT:PSS solvent evaporation due to insufficient interfacial adhesion. The minimum channel length of 20 *μ*m was determined by the receding radius of the droplet. In the current study, with surface modification of the hydrophilic PVA, precise control of the line gap and line width can be realized owing to the pinned contact line^34^. As the result, an ultrashort channel was achieved by direct inkjet printing through careful calculation and adjustment of the printing parameters without any complicated substrate pre-patterning process.

The stable line width through inkjet printing can be estimated by the following equation (1):^35^


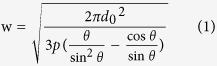


where *d*_0_ is the droplet diameter in air, *θ* is the contact angle of the droplet on the substrate, and *p* is the distance between the consecutive droplets’ landing positions. It’s clear that for a given printer cartridge with the fixed *d*_0_ and *θ*, *p* is the only parameter which can be optimized. To obtain fine lines with smooth edges, *p* can neither be too small nor too large^36^. The optimized *p* for PEDOT:PSS jetted from a 30 *μ*m nozzle, is around 50 *μ*m, which resulted in a line width of about 106 *μ*m without coffee ring (Fig. 2c). Thus, by carefully adjusting the line-center distance between two PEDOT:PSS electrodes, the printed channel length can be determined.

**Figure 3:**
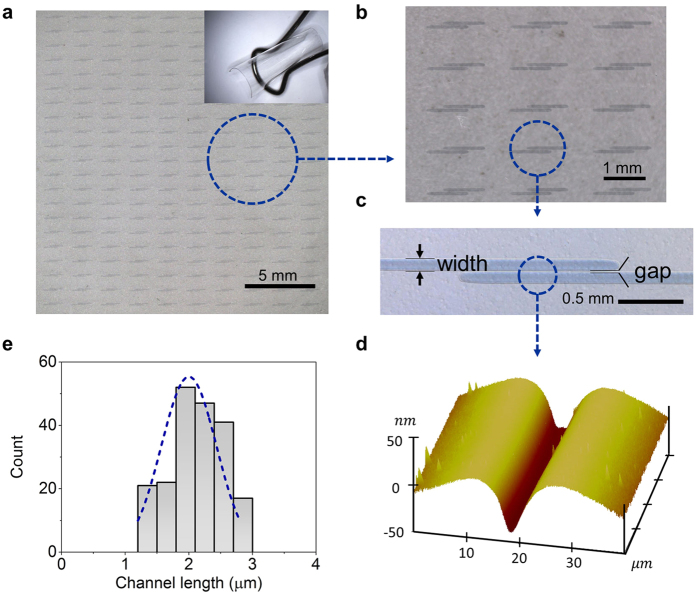
A printed array of ultrashort channels. (**a**) The printed PEDOT:PSS array (10 × 20) on the flexible PET substrate with 2 *μ*m channel length. Inset: Picture of the array on the 3 cm × 3 cm substrate, bended with bending radius of about 5 mm. (**b**) The zoom-in picture of the array. (**c**) Picture of a single channel. (**d**) AFM picture of the PEDOT:PSS lines with a gap of about 2 *μ*m. (**e**) The channel-length distribution of the 200 channels.

By setting the printing line-center distance at 118 *μ*m after taking into account the line width and the substrate positioning accuracy, a 2 *μ*m channel length was successfully achieved. To test the printing uniformity, 200 structures were fabricated on a 3 cm × 3 cm PET substrate with 2 *μ*m channel length and 970 *μ*m channel width. The microscopic pictures and AFM topography of the printed channels are shown in Fig. 3. The inset of Fig. 3a shows that the all-solution processed OTFTs are flexible and transparent. The channel length distribution in Fig. 3e shows the direct inkjet printing is reliable and repeatable (2.0 *μ*m in average with 0.5 *μ*m standard deviation). No shortening of the S/D electrodes was detected confirming the high yield of the device array fabrication.

**Figure 4:**
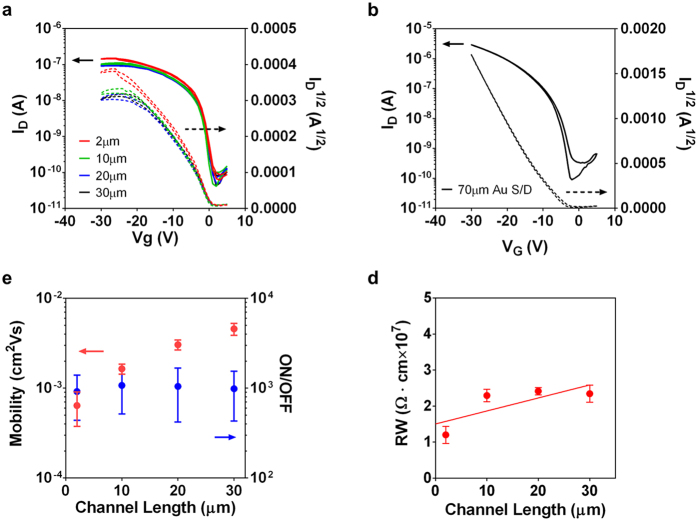
Performance of OTFTs. (**a**) Transfer characteristics of the devices with printed PEDOT:PSS electrodes. The channel width is fixed at 970 *μ*m, and channel lengths are 2 *μ*m, 10 *μ*m, 20 *μ*m and 30 *μ*m, respectively. (**b**) Transfer characteristic of the device with evaporated Au S/D electrodes. W/L = 280 *μ*m/70 *μ*m. (**c**) The dependence of mobility and ON/OFF ratio on the cannel length. (**d**) The channel resistance of devices with different channel lengths (normalized to the channel width). The intercept at the y axis indicates the contact resistance.

The typical transfer characteristics of the 2 *μ*m channel OTFT device is shown in Fig. 4a. The maximum hole mobility is (0.64 ± 0.27) × 10^−3 ^cm^2^/V · s, ON/OFF ratio is (0.92 ± 0.48) × 10^3^, and turn-on voltage is 1.94 ± 0.32 V. The statistics is calculated from a total of 96 devices. For comparison, OTFTs with thermal-evaporated Au S/D electrodes and a channel length of 70 *μ*m (W/L = 4) were fabricated. As shown in Fig. 4b, the devices exhibit a mobility of 0.55 cm^2^/V·s, an ON/OFF ratio of 3.26 × 10^4^, and a turn-on voltage at −5.5 V. A previous study on non-annealed PDQT devices using a bottom-gate top-contact structure with evaporated Au electrodes reported a mobility of 0.89 cm^2^/V·s, an ON/OFF ratio of 10^7^, and a threshold voltage of −2.6 V^37^. The performances of the Au devices indicate that the poor performance of the 2-*μ*m-channel all-organic devices is not due to the semiconducting or the dielectric layer, but the printed PEDOT:PSS electrodes and their interface to the semiconducting layer. It is observed that while the OFF current of 2-*μ*m-channel devices remains at the same level as the Au device, the ON current is about an order of magnitude smaller, which directly reduces the ON/OFF ratio to about 10^3^. It is also noticed that the slope of the square root of drain current quickly decreases as the gate voltage increases. Therefore, it is speculated that the poor performance may be the result of the relatively large contact resistance at the printed electrodes.

To confirm it, all-solution processed, all-organic TFTs with channel lengths of 30 *μ*m, 20 *μ*m and 10 *μ*m were fabricated. The channel width was kept at 970 *μ*m. Transfer characteristics are presented in Fig. 4a,c gives the statistical distribution of the mobility and ON/OFF ratio for the devices with different channel length. The statistics are calculated from a total of 46, 93, and 61 devices, for 30 *μ*m, 20 *μ*m, and 10 *μ*m channel length, respectively. The device performances are summarized in Table 1. The mobility shows a decreasing trend as the channel length shortens (Fig. 4c), which is consistent with the effect of large contact resistance in TFTs^38^. By further plotting the channel resistance (normalized to the channel width) as a function of channel length in Fig. 4d (at the gate voltage of −5 V), a contact resistance of about 15.0 MΩ · cm is identified. The value is about two to three orders of magnitude larger than typical solution-processed OTFTs^39^. Such large contact resistance will limit the drain current at high gate voltages. As a result, calculating mobility from typical equations will lead to much smaller values^38^. The origin of the large contact resistance could come from the relatively large injection barrier from PEDOT:PSS electrodes to the semiconducting PDQT, as well as the large bulk resistance of intrinsic PEDOT:PSS^15^. Further device optimization by, for example, inserting an injection layer between PEDOT:PSS electrodes and the PQDT layer, or raising the conductivity of PEDOT:PSS, will improve the device performance^40^,^41^.

In summary, all-organic and all-solution processed OTFT array is fabricated on flexible substrate PET. The source, drain and gate electrodes are deposited by inkjet printing conducting polymer PEDOT:PSS. By modifying the PET substrate with water-dissolvable PVA, the contact line of PEDOT:PSS is pinned during the drying process resulting in well-defined edges. The pinning effect subsequently enables precise control of the printed channel length. Ultrashort channel of 2 *μ*m is successfully realized without any complicated substrate’s pre-patterning process. No short circuiting was found by printing a large array with 200 electrode pairs. Due to the large contact resistance, the printed devices show inferior performances to those with evaporated metal electrodes. By further reducing the linewidth of the printed patterns as well as optimizing the device performance, application of all-printed, high resolution TFTs in flexible displays, sensor chips, and radio-frequency identification tags will become practical.

## Methods

### Materials

All the chemicals and materials were purchased and used as received unless otherwise noted. PEDOT:PSS Clevios PH 500 was purchased from Heraeus Deutschland GmbH & Co. KG. PVA (98–99% hydrolyzed) was purchased from Alfa Aesar. To make PVA solution (20 mg/mL) in deionized water, the solution was heated to 90 °C for 5 hours. PDQT (molecular weight >30,000) was purchased from Lumtec Corp. PMMA, PEO, and all the solvents were purchased from Aldrich, Inc. All the solutions were filtered through a 0.45 *μ*m filter before use.

### Device Fabrication and Characterization

PEDOT:PSS printing was carried out on an inkjet printer (Jetlab II) from Microfab Technologies, Inc. The printer head’s orifice diameter is 30 *μ*m. Film thickness and surface morphologies were obtained from a Dektak 150 surface profiler (Veeco Instruments, Inc.). The three-dimensional white light interference microscopy pictures were recorded by Veeco Instruments’ NT 9300 surface profiler. The optical microscopic images were taken with a Nikon Eclipse E600 POL with a DXM1200F digital camera. The transistor characteristics of the devices were measured using an Agilent 4155C semiconductor parameter analyzer connected to a Cascade manual probe station.

## Additional Information

**How to cite this article**: Xu, W. *et al*. Flexible All-organic, All-solution Processed Thin Film Transistor Array with Ultrashort Channel. *Sci. Rep*. **6**, 29055; doi: 10.1038/srep29055 (2016).

## Figures and Tables

**Table 1:** Summary of the OTFT performance at different channel length.

Channel length (*μ*m)	Mobility (cm^2^/V s)	ON/OFF	*V*_*turn-on*_ (V)
2	(0.64 ± 0.27)×10^−3^	(0.92 ± 0.48)×10^3^	1.94 ± 0.32
10	(1.64 ± 0.21)×10^−3^	(1.07 ± 0.56)×10^3^	1.93 ± 0.32
20	(3.05 ± 0.39)×10^−3^	(1.05 ± 0.62)×10^3^	1.83 ± 0.13
30	(4.57 ± 0.70)×10^−3^	(0.98 ± 0.55)×10^3^	1.59 ± 0.50
